# Linalool inhibits the angiogenic activity of endothelial cells by downregulating intracellular ATP levels and activating TRPM8

**DOI:** 10.1007/s10456-021-09772-y

**Published:** 2021-03-02

**Authors:** Vivien Becker, Xin Hui, Lisa Nalbach, Emmanuel Ampofo, Peter Lipp, Michael D. Menger, Matthias W. Laschke, Yuan Gu

**Affiliations:** 1 Institute for Clinical & Experimental Surgery, Saarland University, 66421 Homburg, Saarland Germany; 2 Molecular Cell Biology, Research Center for Molecular Imaging and Screening, Medical Faculty, Saarland University, 66421 Homburg, Saarland Germany

**Keywords:** Linalool, Endothelial cells, TRPM8, ATP, Angiogenesis, Vascularization

## Abstract

Angiogenesis crucially contributes to various diseases, such as cancer and diabetic retinopathy. Hence, anti-angiogenic therapy is considered as a powerful strategy against these diseases. Previous studies reported that the acyclic monoterpene linalool exhibits anticancer, anti-inflammatory and anti-oxidative activity. However, the effects of linalool on angiogenesis still remain elusive. Therefore, we investigated the action of (3*R*)-(−)-linalool, a main enantiomer of linalool, on the angiogenic activity of human dermal microvascular endothelial cells (HDMECs) by a panel of angiogenesis assays. Non-cytotoxic doses of linalool significantly inhibited HDMEC proliferation, migration, tube formation and spheroid sprouting. Linalool also suppressed the vascular sprouting from rat aortic rings. In addition, Matrigel plugs containing linalool exhibited a significantly reduced microvessel density 7 days after implantation into BALB/c mice. Mechanistic analyses revealed that linalool promotes the phosphorylation of extracellular signal-regulated kinase (ERK), downregulates the intracellular level of adenosine triphosphate (ATP) and activates the transient receptor potential cation channel subfamily M (melastatin) member (TRPM)8 in HDMECs. Inhibition of ERK signaling, supplementation of ATP and blockade of TRPM8 significantly counteracted linalool-suppressed HDMEC spheroid sprouting. Moreover, ATP supplementation completely reversed linalool-induced ERK phosphorylation. In addition, linalool-induced ERK phosphorylation inhibited the expression of bone morphogenetic protein (BMP)-2 and linalool-induced TRPM8 activation caused the inhibition of β1 integrin/focal adhesion kinase (FAK) signaling. These findings indicate an anti-angiogenic effect of linalool, which is mediated by downregulating intracellular ATP levels and activating TRPM8.

**Supplementary Information:**

The online version contains supplementary material available at 10.1007/s10456-021-09772-y.

## Introduction

Angiogenesis is a highly dynamic process, which is defined as the development of new blood vessels from preexisting ones. During this process, endothelial cells (ECs) are activated by pro-angiogenic factors, such as vascular endothelial growth factor (VEGF) and basic fibroblast growth factor (bFGF), to proliferate, migrate and form new microvascular networks [1]. These activities of ECs are mediated by several pivotal angiogenesis-related pathways, including phosphatidylinositol 3-kinase (PI3K)/AKT/mammalian target of rapamycin (mTOR) and Raf/mitogen-activated protein kinase (MEK)/extracellular signal-regulated kinase (ERK) signaling [2, 3]. Notably, ERK has also been reported to be activated by different stress stimuli, such as oxidative stress and DNA damage stimuli, and suppress cell proliferation and survival [4–7].

Recently, Genova et al. demonstrated an inhibitory effect of transient receptor potential cation channel subfamily M (melastatin) member 8 (TRPM8) on EC adhesion, migration, tube formation and spheroid sprouting [8]. TRPM8, also known as the cold and menthol receptor 1 (CMR1), is the main molecular transducer of cold somatosensation in humans. It can be activated by voltage, cold temperatures and cooling compounds, such as menthol and icilin [9–11]. Upon activation, sodium and calcium ions pass through TRPM8 channels into cells to regulate various cellular processes [12]. Of interest, Genova et al. reported that TRPM8 inhibits EC motility independently of pore function, but retains Rap1 GTPase through direct protein-protein interaction [8].

Angiogenesis occurs during embryogenesis and wound healing. On the other hand, the development of new blood vessels is critically involved in a large number of diseases, including cancer, endometriosis, psoriasis and diabetic retinopathy [13]. Accordingly, the inhibition of angiogenesis represents a promising strategy for the treatment of these diseases. So far, the United States Food and Drug Administration has approved several anti-angiogenic agents targeting the VEGF/VEGF receptor pathway [14]. Unfortunately, these agents are quite expensive and frequently induce severe side effects during long-term treatment. Therefore, there is an urgent need for the development of novel anti-angiogenic drugs with fewer side effects.

Phytochemicals are considered as a rich source for the discovery of new compounds, which are cheap, effective and safe. Such a compound is linalool, a main active ingredient of essential oils of various aromatic species, including lavender, orange and rose. Actually, linalool is produced by over 200 different plant species and also found in some fungi [15]. It is widely used as a fragrance ingredient in shampoos, body lotions, perfume and soaps. Moreover, it is also commonly used as a flavoring in beverages and foods [15]. Previous studies reported a broad spectrum of biological activities for linalool, including sedative, analgesic, anticancer, antioxidant, antimicrobial and anti-inflammatory properties [16, 17]. However, so far nothing is known about its effects on angiogenesis.

Therefore, the aim of the present study was to investigate the action of (3*R*)-(−)-linalool on the proliferation, migration, tube formation and spheroid sprouting of human dermal microvascular endothelial cells (HDMECs). To validate our in vitro findings, we additionally performed an ex vivo aortic ring assay and an in vivo Matrigel plug assay. Moreover, we analyzed the effects of linalool on angiogenesis-related signaling pathways, intracellular levels of adenosine triphosphate (ATP), reactive oxygen species (ROS) formation and TRPM8 activity in HDMECs. In addition, the downstream anti-angiogenic mediators of linalool-induced ATP reduction and TRPM8 activation were identified.

## Materials and methods

### Cell culture and treatment

HDMECs (PromoCell, Heidelberg, Germany) were cultured in endothelial cell growth medium (EGM)-MV (PromoCell) and incubated at 37 °C in a humidified atmosphere containing 5% CO_2_. A stock solution of 5 M (3*R*)-(−)-linalool (Sigma-Aldrich, Darmstadt, Germany) was prepared in dimethyl sulfoxide (DMSO) and further diluted into different concentrations (0.05–5 mM) in EGM-MV. The DMSO concentrations were identical in the linalool- and vehicle-treated groups. The cells were exposed to linalool for 24 h followed by different in vitro assays.

### Cell transfection

To downregulate the expression level of ERK and TRPM8, HDMECs were transfected for 48 h with 20 nM small interfering RNAs (siRNAs) against ERK1/2 (si-ERK; Cell Signaling Technology, Frankfurt, Germany) or 120 nM siRNAs against TRPM8 (si-TRPM8; ON-TARGETplus siRNA SMARTpool, Dharmacon, Colorado, USA) using HiPerFect reagent (Qiagen, Hilden, Germany) according to the manufacturer’s protocol. Negative control of siRNA (si-NC, Qiagen) served as control.

### Water‐soluble tetrazolium (WST)-1 assay

Cell viability was assessed by a WST-1 assay, in which tetrazolium salt is converted into soluble formazan by mitochondrial dehydrogenase. The amount of produced formazan depends on the number of viable cells. As described previously [18], 5 × 10^3^ HDMECs were seeded in each well of 96-well plates and exposed to different compounds for 24 h. Then, 10 µL WST-1 reagent (Roche Diagnostics, Mannheim, Germany) was added into each well and the plate was incubated at 37 °C for 30 min. The absorbance of each well was measured at 450 nm with 620 nm as reference using a microplate photometer (PHOmo; anthos Mikrosysteme GmbH, Krefeld, Germany).

### Lactate dehydrogenase (LDH) assay

The cytotoxicity of linalool was analyzed by a LDH assay according to the manufacturer’s instructions (Roche Diagnostics). Briefly, 5 × 10^3^ HDMECs were seeded in each well of 96-well plates and exposed for 24 h to different concentrations of linalool. Then, 100 µL LDH reaction mix was added into each well. After 10 min of incubation at room temperature, the reaction was stopped by addition of 50 µL stop solution. The absorbance of each well was then measured at 492 nm with 620 nm as reference using a microplate photometer (PHOmo).

### Flow cytometry

The effects of linalool on EC proliferation, ROS formation and β1 integrin activation were analyzed by means of flow cytometry.

To assess cell proliferation, a bromodeoxyuridine (BrdU) assay was performed. Briefly, HDMECs seeded in 60-mm dishes were exposed to different concentrations of linalool for 6 h followed by BrdU addition and incubation for another 18 h. Then, the cells were fixed with 70% ethanol for 30 min on ice and incubated with 2 M hydrochloric acid (HCl) containing 0.5% Triton-X 100 for 30 min at room temperature for DNA denaturation. After incubation with a fluorescein isothiocyanate (FITC)-labeled anti-BrdU antibody (1:50; Thermo Fisher Scientific, Karlsruhe, Germany) for 30 min, the cells were measured with a FACScan flow cytometer (BD Biosciences, Heidelberg, Germany).

Intracellular ROS levels were measured with 2′,7′-dichloro-dihydro-fluorescein diacetate (DCFH-DA). Briefly, 3 × 10^5^ HDMECs seeded in 6-well plates were pretreated for 2 h with 0.5 mM reducing agent dithiothreitol (DTT) and then exposed for 24 h to 0 or 2 mM linalool. Then, the cells were incubated with 0.5 µM DCFH-DA (Sigma-Aldrich) at 37 °C for 30 min. Alternatively, HDMECs were pretreated for 2 h with 0.5 mM DTT, incubated for 30 min with 0.5 µM DCFH-DA and then treated for 30 min with 0.5 mM H_2_O_2_. After collecting the cells with a scraper, the intracellular fluorescence intensity was then measured by a FACScan flow cytometer (BD Biosciences). H_2_O_2_-treated cells served as a positive control for ROS generation.

To evaluate the activation of β1 integrin, 3 × 10^5^ HDMECs seeded in 6-well plates were pretreated for 2 h with or without 5 µM *N*-(3-aminopropyl)-2-[(3-methylphenyl) methoxy]-*N*-(2-thienylmethyl)-benzamide hydrochloride (AMTB, Sigma-Aldrich), a specific TRPM8 inhibitor. Then, the cells were exposed to 2 mM DMSO or linalool for 30 min, followed by rinse with HEPES buffer supplemented with 1 mM Mn^2+^ and incubation with PE-conjugated anti-β1 integrin antibody (clone HUTS-21; 1:20; BD Pharmingen, Heidelberg, Germany) at 37 °C for 30 min. After collection with a cell scraper, the cells were measured with a FACScan flow cytometer (BD Biosciences).

### Migration assay

EC migration was analyzed by two different assays. For the scratch wound healing assay, 3.2 × 10^5^ HDMECs were seeded in 35-mm dishes and then exposed to different concentrations of linalool. After 24 h of incubation, four scratches were generated onto the cell monolayer using a 10 µL pipette tip. Immediately after scratching (0 h) and after 6 h, images of the scratches were taken using a phase-contrast microscope (Leica DFC450C;  Leica Microsystems, Wetzlar, Germany). Wound areas were measured using the LAS V4.8 software (Leica Microsystems).

For the transwell migration assay, HDMECs were seeded in 100-mm dishes and then exposed for 24 h to different concentrations of linalool. Thereafter, 2.5 × 10^5^ HDMECs treated with linalool or vehicle were suspended in 500 µL endothelial basal medium (EBM) without supplements and seeded into the inserts of 24-transwell plates (8 µm pores; Corning, Wiesbaden, Germany), while 750 µL EBM containing 1% fetal calf serum (FCS) was added to the bottom chamber. After 5 h of incubation, the non-migrated cells were removed and the migrated cells were stained with Diff-Quick (LT-SYS, Berlin, Germany) and counted under a BZ-8000 microscope (Keyence, Osaka, Japan).

### Tube formation assay

To assess the tube-forming activity of ECs, 1.7 × 10^4^ HDMECs were seeded into each well of 96-well plates pre-coated with 50 µL Matrigel (Corning) and then treated with different concentrations of linalool. After 24 h, the newly formed vessel-like structures were observed under a phase-contrast microscope (BZ-8000; Keyence) and analyzed by quantifying the number of meshes using ImageJ software with the angiogenesis analyzer plug-in [U.S. National Institutes of Health (NIH), Bethesda, Maryland, USA].

### Spheroid sprouting assay

As previously described [19], 500 HDMECs suspended in EGM-MV containing 20% methylcellulose (w/v) (Thermo Fisher Scientific) were seeded into each well of non-adherent round bottom 96-well plates. After 24 h of incubation, spheroids were collected and suspended in a collagen solution mixed with EBM containing 20% FCS and 0.5% methylcellulose (w/v) at a ratio of 1:2. This collagen solution consists of 8 volumes rat acidic collagen extract (Serva, Heidelberg, Germany), 1 volume 10 × Medium 199 (Sigma-Aldrich) and 1 volume 0.2 M NaOH. Then, the spheroid mixture was rapidly transferred into a pre-warmed 24-well plate. After incubation for 45 min, EGM-MV containing different concentrations of linalool was gently added onto the spheroids. The spheroids were photographed after 24 h of incubation by a phase-contrast microscope (Leica DFC450C) and the cumulative length of the sprouts that had grown out of each spheroid was measured using the LAS V4.8 software (Leica Microsystems).

### Aortic ring assay

As previously described [20], aortic rings from Wistar albino Glaxo rats were embedded in 200 µL Matrigel (Corning) in each well of 48-well plates. After incubation for 15 min allowing for Matrigel polymerization, 800 µL Dulbecco’s modified Eagle’s medium (DMEM) containing 10% FCS, penicillin, streptomycin and different concentrations of linalool was added. After 6 days with a medium change on day 3, the aortic rings were photographed by a phase-contrast microscope (BZ-8000; Keyence) and the area of vascular sprouting from the aortic rings was measured using the image analysis application software (Keyence).

### In vivo Matrigel plug assay

The in vivo effects of linalool on angiogenesis were evaluated in a Matrigel plug assay. Briefly, 200 µL growth factor-reduced Matrigel (Corning) containing 30 IU/mL heparin (Braun, Melsungen, Germany), 1 µg/mL VEGF (PAN Biotech, Bayern, Germany) and 2 mM DMSO or linalool was injected subcutaneously into 5–7-month-old male BALB/c mice. After 7 days, the Matrigel plugs were excised for immunohistochemical analyses. This experiment was approved by the Local Animal Protection Committee and was conducted according to the German legislation for animal welfare and the Guide for the Care and Use of Laboratory Animals (8th Edition, 2011).

### Immunohistochemistry

For the detection of microvessels within the Matrigel plugs, sections were cut and stained with a rat anti-mouse CD31 antibody (1:100; Dianova, Hamburg, Germany) followed by a goat-anti-rat Alexa Fluor 555-labeled secondary antibody (1:100; Life Technologies). Cell nuclei were visualized by staining with Hoechst 33342 (Sigma-Aldrich). Sections were then analyzed using a fluorescence microscope (BZ-8000; Keyence) and the microvessel density was determined by counting the number of CD31-positive microvessels in 6 microscopic regions of interest (ROIs) of each plug.

### Western blot analysis

Treated HDMECs were lysed with lysis buffer composed of 10 mM Tris (pH 7.5), 10 mM NaCl, 0.1 mM EDTA, 0.5% Triton-X 100, 0.02% NaN_3_, phenylmethylsulfonyl fluoride (1:500 v/v), protease inhibitor cocktail (1:100 v/v; Sigma-Aldrich) and phosphatase inhibitor cocktail (1:100 v/v; Sigma-Aldrich) for 10 min on ice. The cell lysate was then transferred to tubes and centrifuged at 4 °C for 30 min at 13,000 × *g*. The supernatant was collected and protein concentrations were measured using the Pierce BCA Protein Assay (Thermo Fisher Scientific) with BSA as a standard. Subsequently, 10 µg proteins per lane were separated on 10% sodium dodecyl sulfate (SDS) polyacrylamide gels and transferred to a polyvinylidene difluoride (PVDF) membrane (BioRad, Munich, Germany). The membrane was then blocked with Blotting-Grade Blocker (BioRad) and incubated with a rabbit monoclonal anti-pAKT1/2/3 antibody (1:100; Cell Signaling Technology), a rabbit monoclonal anti-AKT antibody (1:500; Cell Signaling Technology), a mouse monoclonal anti-pERK antibody (1:300; Abcam, Cambridge, UK), a rabbit polyclonal anti-ERK antibody (1:300; Abcam), a rabbit monoclonal anti-focal adhesion kinase (FAK) antibody (1:100; Cell Signaling Technology), a rabbit monoclonal anti-pFAK antibody (1:250; Cell Signaling Technology), a rabbit monoclonal anti-TRPM8 antibody (1:25; Abcam), a mouse monoclonal anti-bone morphogenetic protein (BMP)-2 antibody (1:30; Proteintech, Manchester, UK), a rabbit polyclonal anti-cyclin-dependent kinase (CDK)4 antibody (1:10; Santa Cruz Biotechnology, Texas, USA), a rabbit polyclonal anti-CDK6 antibody (1:30; Santa Cruz Biotechnology), a rabbit monoclonal anti-CDK9 antibody (1:150; Cell Signaling Technology), a rabbit polyclonal anti-cyclooxygenase (COX)-2 antibody (1:50; Abcam), a mouse monoclonal anti-endothelial nitric oxide synthase (eNOS) antibody (1:100; BD Bioscience), a mouse monoclonal anti-intercellular adhesion molecule (ICAM)-1 antibody (1:30; Santa Cruz Biotechnology), a mouse monoclonal anti-p21 antibody (1:250; Abcam), a mouse monoclonal anti-Ras homolog family member A (RhoA) antibody (1:250; Cell Signalling Technology), a mouse monoclonal anti-sirtuin (SIRT)1 antibody (1:100; Abcam), a rabbit polyclonal anti-VEGFA antibody (1:50; Abcam) or a mouse monoclonal anti-β-actin antibody (1:5000; Sigma-Aldrich), followed by an anti-mouse (1:1500; Dako/Agilent, Hamburg, Germany) or anti-rabbit secondary antibody conjugated to horseradish peroxidase (HRP) (1:1000; R&D, Abingdon, UK). Protein signals were detected with an enhanced chemiluminescence (ECL) kit (GE Healthcare, Freiburg, Germany) under a Chemocam device (Intas, Göttingen, Germany) and the band intensities were analyzed using ImageJ software (NIH).

### Intracellular ATP measurement

The intracellular ATP level was measured using an ATP Determination Kit (Thermo Fisher Scientific) according to the manufacturer’s instructions. Briefly, HDMECs were exposed to 2 mM DMSO or linalool in the presence or absence of 5 µM AMTB (Sigma-Aldrich) or 1 µM PD0325901, which specifically inhibits MEK/ERK signaling (Selleckchem, Munich, Germany). After 24 h of incubation, the cells were rinsed with phosphate-buffered saline (PBS) and lysed with lysis buffer (Promega, Walldorf, Germany) for 15 min at room temperature by shaking. The cell lysate was then centrifuged with full speed for 5 min. The supernatant was collected and mixed with standard reaction solution consisting of reaction buffer, 0.1 M DTT, 10 mM d-luciferin and firefly luciferase. Luminescence was measured using a Tecan Infinite M200 Pro luminometer (Männedorf, Switzerland).

### Ca^2+^ transient acquisition and analysis

Ca^2+^ measurements in living ECs were conducted as previously described [21]. Briefly, 1 × 10^5^ HDMECs were seeded on a coverslip in each well of 12-well plates and treated for 2 h with 5 µM AMTB, 1 µM PD0325901 or 2.5 µM BAPTA-AM (Abcam), which is a specific Ca^2+^ chelator. After loading with Fluo-4 AM (Life Technologies), cells were bathed in Tyrode’s solution (135 mM NaCl, 5.4 mM KCl, 1.8 mM CaCl_2_, 10 mM glucose, 2 mM MgCl_2_ and 10 mM HEPES; pH 7.35) at 26 °C and mounted on an inverted microscope (Eclipse TE, Nikon, Düsseldorf, Germany) with 20x oil-immersion objectives (Plan Fluor, 20 × 0.75, Nikon). Excitation was performed at the wavelength of 470 nm by LED (pE-100, CoolLED Ltd., Andover, USA) and the emission of Fluo-4 was detected by a COMS camera (Orca Flash 4.0; Hamamatsu, Japan) with imaging at 5 frames per second in a size of 512 × 512 pixels. Stimulation with chemical compounds at indicated concentrations was achieved by a local solenoid-controlled gravity-driven perfusion system. The acquired images were analyzed by ImageJ software to correct the image background and collect the fluorescence intensity in ROIs over time. The data were imported into IgorPro (Wavemetrics, Lake Oswego, USA) and processed as the self-ratio traces (*F*/*F*_0_), for which the fluorescence at any given time point (*F*) was divided by the resting fluorescence (*F*_0_) to account for different dye loading. The amplitude of Ca^2+^ transient was calculated as the peak value of the self-ratio trace.

### Statistics

Statistical analysis was performed using the SigmaPlot software (SigmaStat; Jandel Corporation, San Rafael, CA, USA). Differences between two groups were analyzed by the unpaired Student’s *t*-test and differences between multiple groups were analyzed by one-way ANOVA followed by the Student–Newman–Keuls post hoc test including Bonferroni correction to compensate for multiple comparisons. All values were expressed as means ± SEM. Statistical significance was accepted for *P*-values < 0.05.

## Results

### Action of linalool on HDMEC viability and proliferation

**Fig. 1:**
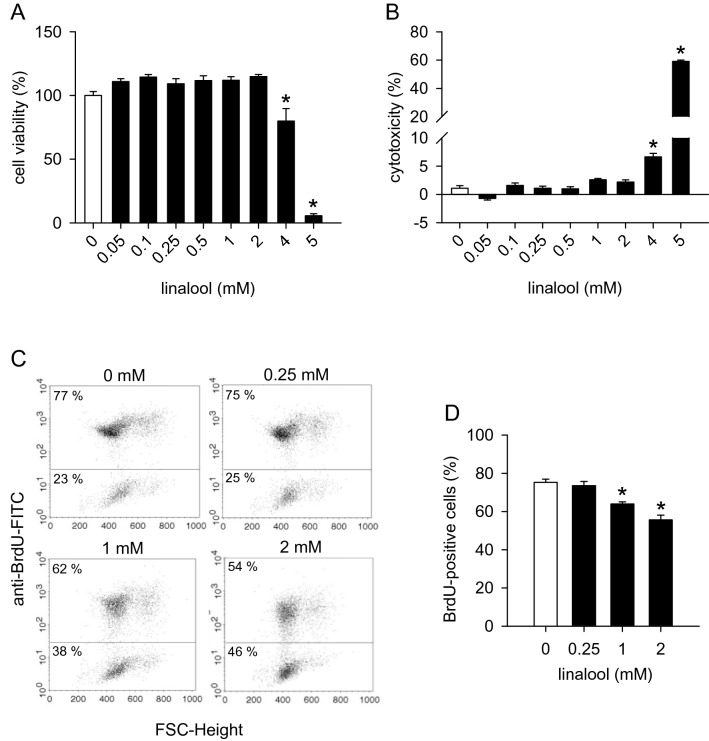
Linalool inhibits HDMEC proliferation. **a**, **b** Viability (in % of 0 mM) of HDMECs (**a**) and cytotoxicity (in % of the total cell death) of linalool (**b**) as assessed by the WST-1 (**a**) and LDH assay (**b**), respectively. HDMECs were exposed for 24 h to serial dilutions (0.05–5 mM) of linalool or vehicle (0 mM) (*n* = 4). **c**: Flow cytometry scatter plots of HDMECs that were exposed for 24 h to 0.25, 1 and 2 mM linalool or vehicle (0 mM), incorporated with BrdU and then stained with FITC-conjugated anti-BrdU antibody. **d** BrdU-positive cells (in % of the total cell number) of vehicle- or linalool-treated HDMECs (*n* = 3). Means ± SEM. *P < 0.05 vs. 0 mM

We first investigated the effects of linalool on HDMEC viability and cytotoxicity by means of WST-1 and LDH assays, respectively. These assays showed that linalool concentrations of up to 2 mM do not affect the viability of HDMECs (Fig. 1a) and do not exert any cytotoxic effects on these cells (Fig. 1b). Accordingly, we chose non-cytotoxic concentrations between 0.25 and 2 mM linalool for our in vitro and in vivo angiogenesis assays.

Next, we analyzed the effects of linalool on HDMEC proliferation. For this purpose, BrdU incorporated into cellular DNA during cell proliferation was detected by flow cytometry. We found that 1 and 2 mM linalool causes a 15% and 23% reduction in HDMEC proliferation, respectively (Fig. 1c, d).

### Action of linalool on HDMEC migration

**Fig. 2:**
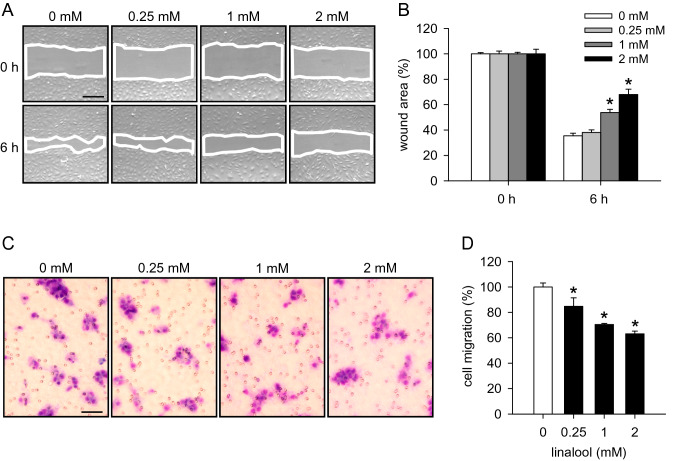
Linalool inhibits HDMEC migration. **a** Phase-contrast microscopic images of HDMEC monolayers, which were scratched and then exposed for 6 h to 0.25, 1 and 2 mM linalool or vehicle (0 mM). Wound areas are marked with white lines. Scale bar 220 µm. **b** Wound area (in % of 0 h) of scratched HDMEC monolayers at 0 and 6 h, as assessed by the scratch wound healing assay (*n* = 8). **c** Light microscopic images of migrated HDMECs, which were treated for 24 h with 0.25, 1 and 2 mM linalool or vehicle (0 mM). Scale bar 70 µm. **d** Migration (in % of 0 mM) of HDMECs, which were treated with vehicle or linalool, as assessed by the transwell migration assay (*n* = 4). Means ± SEM. *P < 0.05 vs. 0 mM

To study the effects of linalool on the migratory activity of HDMECs, scratch wound healing and transwell migration assays were performed. Linalool significantly delayed the wound closure of scratched HDMEC monolayers (Fig. 2a, b) and dose-dependently reduced the number of migrated HDMECs (Fig. 2c, d).

### Action of linalool on HDMEC tube formation and spheroid sprouting

**Fig. 3:**
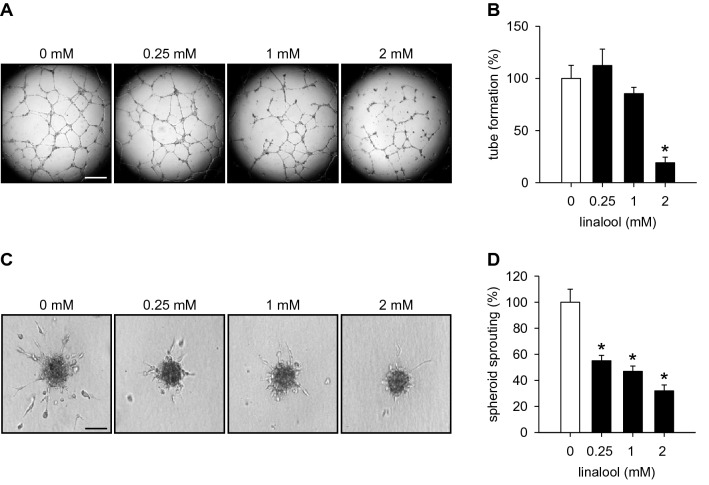
Linalool suppresses HDMEC tube formation and spheroid sprouting. **a** Phase-contrast microscopic images of tube-forming HDMECs. The cells were exposed for 18 h to 0.25, 1 and 2 mM linalool or vehicle (0 mM). Scale bar 750 µm. **b** Tube formation (in % of 0 mM) of HDMECs, which were treated with vehicle or linalool, as assessed by the tube formation assay (*n* = 4). **c** Phase-contrast microscopic images of HDMEC spheroids, which were treated for 24 h with 0.25, 1 and 2 mM linalool or vehicle (0 mM). Scale bar 90 µm. **d** Sprouting (in % of 0 mM) of HDMEC spheroids, which were treated with vehicle or linalool, as assessed by the spheroid sprouting assay (*n* = 15). Means ± SEM. *P < 0.05 vs. 0 mM

We then analyzed the effects of linalool on the tube-forming capacity of HDMECs. Treatment with 2 mM linalool significantly reduced the number of newly formed tube meshes in a tube formation assay (Fig. 3a, b).

Moreover, we demonstrated in a three-dimensional spheroid sprouting assay that linalool dose-dependently diminishes the cumulative length of sprouts growing out of HDMEC spheroids (Fig. 3c, d).

### Action of linalool on angiogenesis ex vivo and in vivo

**Fig. 4:**
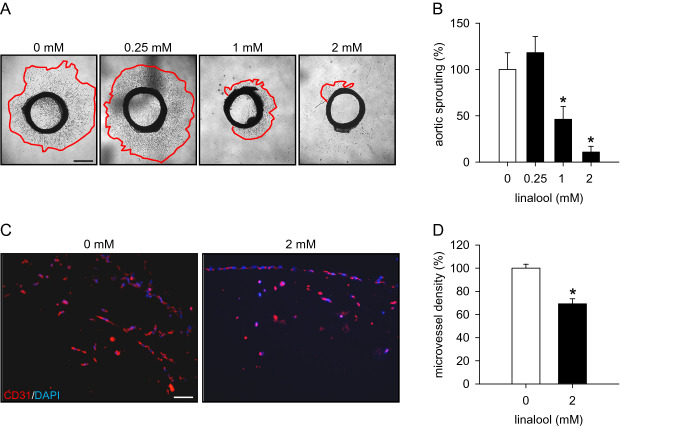
Linalool inhibits angiogenesis ex vivo and in vivo. **a** Phase-contrast microscopic images of rat aortic rings, which were embedded in Matrigel and then exposed to 0.25, 1 and 2 mM linalool or vehicle (0 mM) for 6 days. Scale bar 750 µm. **b** Sprouting (in % of 0 mM) of rat aortic rings, which were treated with vehicle or linalool, as assessed by the aortic ring assay (*n* = 11). **c** Fluorescence microscopic images of sections from Matrigel plugs containing 0 or 2 mM linalool. The sections were stained with anti-mouse CD31 antibody (red) and Hoechst 33342 (blue) to visualize ECs and cell nuclei, respectively. Scale bar 45 µm. **d** Microvessel density (in % of 0 mM) of Matrigel plugs containing 0 or 2 mM linalool, as assessed by immunohistochemistry (*n* = 9). Means ± SEM. *P < 0.05 vs. 0 mM

To confirm our in vitro findings, we performed an ex vivo aortic ring assay. In this assay, rat aortic rings embedded in Matrigel were incubated with different doses of linalool for 6 days. By this, we observed that 1 and 2 mM linalool significantly decreases the area of sprouting from aortic rings when compared to vehicle-treated controls (Fig. 4a, b).

In addition, we evaluated the effects of linalool on angiogenesis in an in vivo Matrigel plug assay. Matrigel plugs containing 2 mM linalool exhibited a 31% lower microvessel density at day 7 after implantation into BALB/c mice when compared to controls (Fig. 4c, d).

### Action of linalool on AKT and ERK signaling

**Fig. 5:**
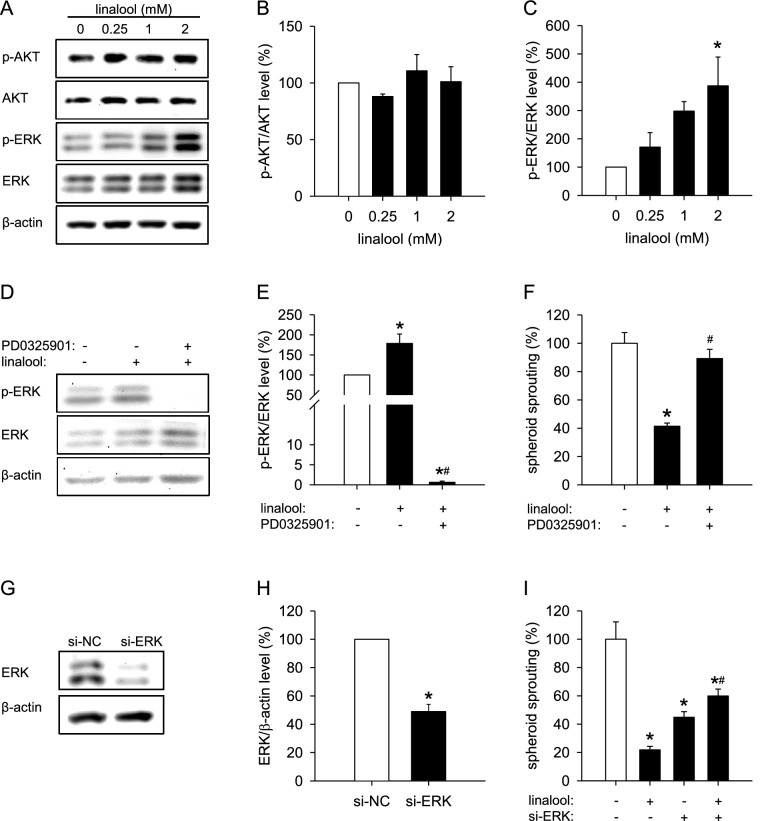
Linalool inhibits angiogenesis through activation of ERK. **a** Western blot of p-AKT, AKT, p-ERK, ERK and β-actin expression in HDMECs, which were exposed for 30 min to 0.25, 1 and 2 mM linalool or vehicle (0 mM). **b**, **c** Expression levels of pAKT/AKT (**b**) and pERK/ERK (**c**) (in % of 0 mM linalool) as assessed by western blot (*n* = 3 independent experiments). **d** Western blot of p-ERK, ERK and β-actin expression in HDMECs, which were pretreated for 2 h with or without 1 µM PD0325901, a specific MEK inhibitor, and then exposed for 30 min to 0 or 2 mM linalool. **e** Expression levels of pERK/ERK (in % of 0 mM linalool) as assessed by western blot (*n* = 4 independent experiments). **f** Sprouting (in % of 0 mM linalool) of HDMEC spheroids, which were treated for 24 h with 0 or 2 mM linalool in the presence or absence of 1 µM PD0325901, as assessed by the spheroid sprouting assay (*n* = 10). **g** Western blot of ERK and β-actin expression in HDMECs, which were transfected for 48 h with 20 nM si-NC or si-ERK. **h** Expression levels of ERK/β-actin (in % of si-NC) as assessed by western blot (*n* = 3 independent experiments). **i** Sprouting (in % of 0 mM linalool) of HDMEC spheroids consisting of si-NC- or si-ERK-transfected cells, which were treated for 24 h with 0 or 2 mM linalool, as assessed by the spheroid sprouting assay (*n* = 10). Means ± SEM. *P < 0.05 vs. 0 mM linalool or si-NC. ^#^*P* < 0.05 vs. 2 mM linalool

To clarify the molecular mechanisms underlying the inhibitory action of linalool on angiogenesis, we first analyzed the activity of two pivotal angiogenic signaling pathways, i.e., PI3K/AKT/mTOR and Raf/MEK/ERK, in HDMECs exposed to different concentrations of linalool. Western blot analyses revealed that linalool strongly increases the phosphorylation of ERK without affecting AKT phosphorylation (Fig. 5a–c). Based on this result, we blocked the ERK pathway with PD0325901, a highly selective and non-ATP-competitive MEK inhibitor. For this purpose, we used a concentration of 1 µM PD0325901, which was non-cytotoxic in a WST-1 assay (Fig. S1a), but reduced the linalool-increased as well as the basal level of phosphorylated ERK (Fig. 5d, e). Of interest, the inhibition of ERK phosphorylation markedly counteracted linalool-suppressed HDMEC spheroid sprouting (Figs. 5f, S2a). To further confirm this finding, we downregulated the intracellular level of ERK by transfecting HDMECs with siRNAs (si-ERK). The transfection efficiency of si-ERK was assessed by western blot and is shown in Fig. 5g, h. As expected, downregulation of ERK significantly suppressed the sprouting of HDMEC spheroids (Figs. 5i, S2b). However, ERK knockdown counteracted linalool-suppressed spheroid sprouting (Figs. 5i, S2b). This indicates that linalool inhibits angiogenesis at least partially by promoting ERK phosphorylation.

### Action of linalool on intracellular ROS levels

**Fig. 6:**
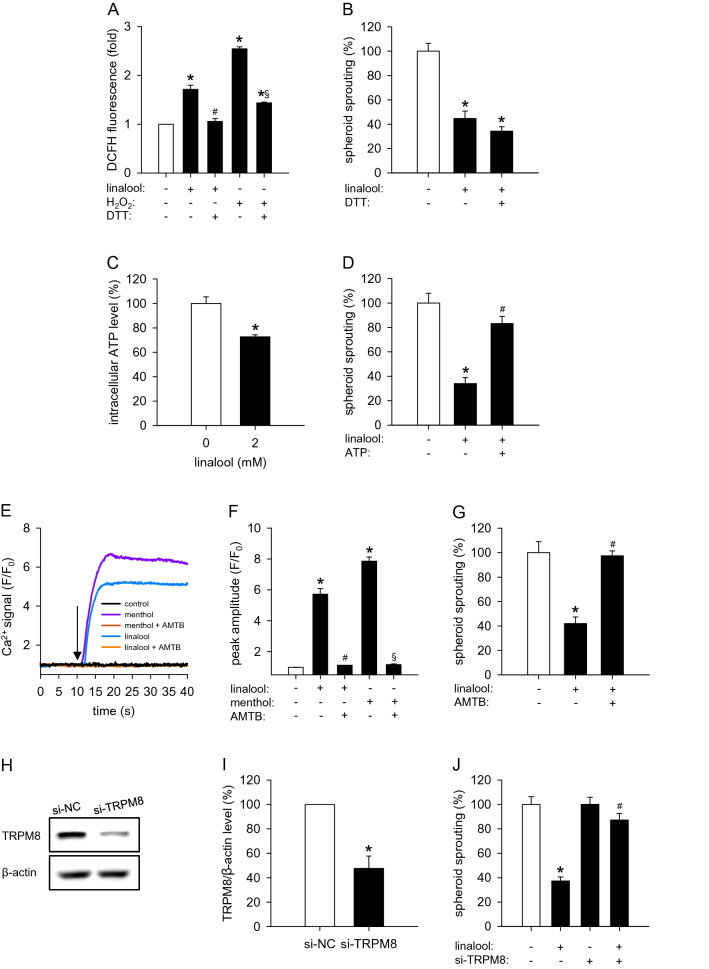
Linalool inhibits angiogenesis through reducing intracellular ATP and activating TRPM8. **a** DCFH fluorescence (in fold of 0 mM linalool) in HDMECs, which were pretreated with or without 0.5 mM DTT, a reducing agent, and then exposed to 0 or 2 mM linalool for 24 h or 0.5 mM H_2_O_2_ for 30 min, as assessed by flow cytometry (*n* = 3 independent experiments). **b** Sprouting (in % of 0 mM linalool) of HDMEC spheroids, which were treated for 24 h with 0 or 2 mM linalool in the presence or absence of 0.5 mM DTT, as assessed by the spheroid sprouting assay (*n* = 10). **c** Intracellular ATP level (in % of 0 mM) of HDMECs, which were treated for 24 h with 0 or 2 mM linalool, as assessed by the luciferase assay (*n* = 4). **d** Sprouting (in % of 0 mM linalool) of HDMEC spheroids, which were treated for 24 h with 0 or 2 mM linalool in the presence or absence of 1 mM ATP, as assessed by the spheroid sprouting assay (*n* = 10). **e** Ca^2+^ signal (*F*/*F*_0_) in HDMECs, which were pretreated with or without 5 µM AMTB (a specific TRPM8 blocker) for 2 h, loaded with Fluo-4 AM and then stimulated with 0 or 2 mM linalool or 1 mM menthol. The onset of stimulation is indicated by a black arrow. **f** Peak amplitude (*F*/*F*_0_) of Ca^2+^ signal in HDMECs, which were pretreated with or without AMTB, loaded with Fluo-4 AM and then stimulated with vehicle, linalool or menthol. **g** Sprouting (in % of 0 mM linalool) of HDMEC spheroids, which were treated for 24 h with 0 or 2 mM linalool in the presence or absence of 5 µM AMTB, as assessed by the spheroid sprouting assay (*n* = 10). **h** Western blot of TRPM8 and β-actin expression in HDMECs, which were transfected for 48 h with 120 nM si-NC or si-TRPM8. **i** Expression levels of TRPM8/β-actin (in % of si-NC) as assessed by western blot (*n* = 3 independent experiments). **j** Sprouting (in % of 0 mM linalool) of HDMEC spheroids consisting of si-NC- or si-TRPM8-transfected cells, which were treated for 24 h with 0 or 2 mM linalool, as assessed by the spheroid sprouting assay (*n* = 10). Means ± SEM. *P < 0.05 vs. 0 mM linalool or si-NC. ^#^*P* < 0.05 vs. 2 mM linalool. ^§^*P* < 0.05 vs. H_2_O_2_ or menthol

Recent studies showed that linalool induces ROS formation in several types of tumor cells, including glioblastoma U87 and hepatocellular carcinoma HepG2 cells [22, 23]. Uncontrolled ROS formation has been reported to impair the activity of ECs [24]. Indeed, plenty of chemical compounds, such as thalidomide, magnolol and dextran-catechin, exert their anti-angiogenic effects by triggering ROS production [25–27]. Therefore, we assessed the effects of linalool on the intracellular ROS levels of ECs. By flow cytometry following DCFH-DA staining, we observed a significant increase of ROS production in linalool-treated HDMECs when compared to vehicle-treated controls (Fig. 6a). H_2_O_2_ was used as a positive control for ROS generation.

To further investigate whether ROS formation contributes to the inhibitory effect of linalool on angiogenesis, we utilized DTT, a wildly used reducing agent, to inhibit the production of ROS. For this purpose, linalool-exposed HDMEC spheroids were treated with a concentration of 0.5 mM DTT, which was non-cytotoxic (Fig. S1c), but effectively suppressed linalool- and H_2_O_2_-induced ROS production (Fig. 6a). However, our data showed that DTT has no effects on linalool-suppressed HDMEC sprouting (Figs. 6b, S3a).

### Action of linalool on intracellular ATP levels

A previous study reported that linalool decreases intracellular ATP levels and reduces the viability of HepG2 cells by inhibiting the mitochondrial complexes I and II [28]. Given the fact that ATP, the primary energy storage molecule, is essential for EC survival, proliferation and migration, we measured the intracellular ATP level of HDMECs by means of a luciferase assay. Treatment with 2 mM linalool significantly reduced the intracellular ATP level by 28% when compared to vehicle-treated controls (Fig. 6c). Based on these findings, we added exogenous ATP to HDMEC spheroids. This efficiently rescued the inhibition of HDMEC spheroid sprouting induced by linalool (Figs. 6d, S3b), indicating that linalool inhibits angiogenesis at least partially through decreasing intracellular ATP levels.

### Action of linalool on TRPM8 activity

Linalool was identified by Behrendt et al. as an agonist of TRPM8 [29]. This calcium-permeable cation channel has recently been shown to inhibit EC adhesion, migration and tube formation [8]. Therefore, we analyzed the activity of TRPM8 in linalool-treated HDMECs by measuring the intracellular calcium concentration. Menthol, a well-known TRPM8 agonist, highly stimulated the influx of Ca^2+^ into HDMECs (Fig. 6e, f). Similarly, linalool markedly increased intracellular Ca^2+^ concentrations (Fig. 6e, f). To further investigate whether linalool evokes Ca^2+^ influx through activating TRPM8, we utilized the specific TRPM8 inhibitor AMTB. By means of a WST-1 assay, we observed that 10–100 µM AMTB significantly reduces HDMEC viability (Fig. S1b). Accordingly, we chose a non-cytotoxic concentration of 5 µM AMTB for the following assays. As expected, inhibition of TRPM8 with AMTB completely blocked the menthol-stimulated Ca^2+^ influx (Fig. 6e, f). Likewise, AMTB also totally reversed the increased intracellular Ca^2+^ concentration in linalool-treated HDMECs (Fig. 6e, f), indicating that linalool activates TRPM8 in these cells.

To analyze whether activated TRPM8 mediates the anti-angiogenic activity of linalool, the specific TRPM8 inhibitor AMTB and siRNAs against TRPM8 (si-TRPM8) were exploited in the spheroid sprouting assays. The transfection efficiency of si-TRPM8 is evaluated in Fig. 6h, i. Consistently, both TRPM8 inhibition and knockdown completely reversed linalool-reduced HDMEC spheroid sprouting (Figs. 6g, j, S3c, d), indicating that linalool downregulates angiogenesis at least in part through activating TRPM8.

### Relation between ERK phosphorylation, intracellular ATP reduction and TRPM8 activation

**Fig. 7:**
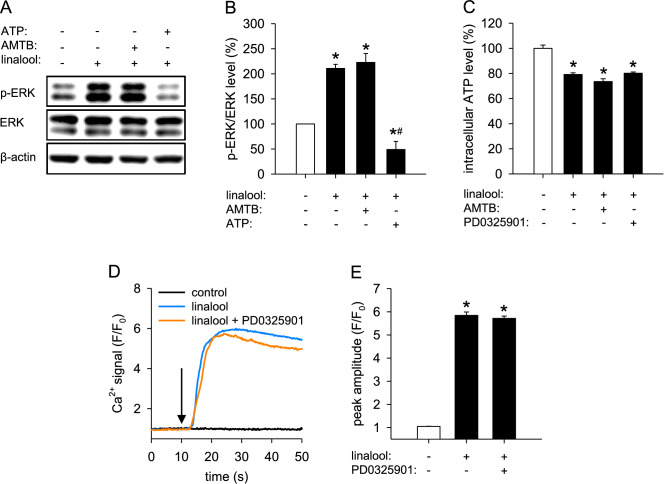
Supplementation of ATP reverses linalool-induced ERK phosphorylation independently of TRPM8 activation. **a** Western blot of p-ERK, ERK and β-actin expression in HDMECs, which were pretreated for 2 h with or without 5 µM AMTB or 1 mM ATP and then exposed for 30 min to 0 or 2 mM linalool. **b** Expression levels of pERK/ERK (in % of 0 mM linalool) as assessed by western blot (*n* = 3 independent experiments). **c** Intracellular ATP level (in % of 0 mM linalool) of HDMECs, which were treated with 0 or 2 mM linalool in the presence or absence of 5 µM AMTB or 1 µM PD0325901, as assessed by the luciferase assay (*n* = 4). **d** Ca^2+^ signal (*F*/*F*_0_) in HDMECs, which were pretreated for 2 h with or without 1 µM PD0325901, loaded with Fluo-4 AM and then stimulated with 0 or 2 mM linalool. The onset of stimulation is indicated by a black arrow. **e** Peak amplitude (*F*/*F*_0_) of Ca^2+^ signal in HDMECs, which were pretreated with or without PD0325901, loaded with Fluo-4 AM and then stimulated with vehicle or linalool. Means ± SEM. *P < 0.05 vs. 0 mM linalool. ^#^*P* < 0.05 vs. 2 mM linalool

The findings above demonstrate that linalool inhibits angiogenesis through upregulating the phosphorylation of ERK, reducing intracellular ATP levels and activating TRPM8. To determine the relation between these three mechanisms, we assessed the phosphorylation of ERK in HDMECs that were pretreated with AMTB or ATP and then exposed to linalool. Western blot analyses showed that the pretreatment with ATP, but not with AMTB, completely reversed linalool-induced ERK phosphorylation (Fig. 7a, b). This result suggests that the linalool-increased ERK phosphorylation is caused by the decreased intracellular ATP levels, independently of TRPM8 activation.

Furthermore, we checked the intracellular ATP levels of HDMECs treated with linalool alone or in combination with AMTB or PD0325901. Luciferase assays showed that neither AMTB nor PD0325901 affects the reduction of intracellular ATP levels induced by linalool (Fig. 7c). These data confirm that intracellular ATP reduction locates upstream of ERK phosphorylation and also indicate that linalool-induced intracellular ATP reduction is independent of TRPM8 activation.

In addition, we assessed the activity of TRPM8 in HDMECs that were pretreated with PD0325901 and then exposed to linalool. Intracellular Ca^2+^ measurements revealed that PD0325901 has no effect on linalool-stimulated Ca^2+^ influx (Fig. 7d, e). This demonstrates that linalool activates TRPM8 independently of ERK phosphorylation.

### Downstream anti‐angiogenic mediators of linalool‐induced ERK phosphorylation

**Fig. 8:**
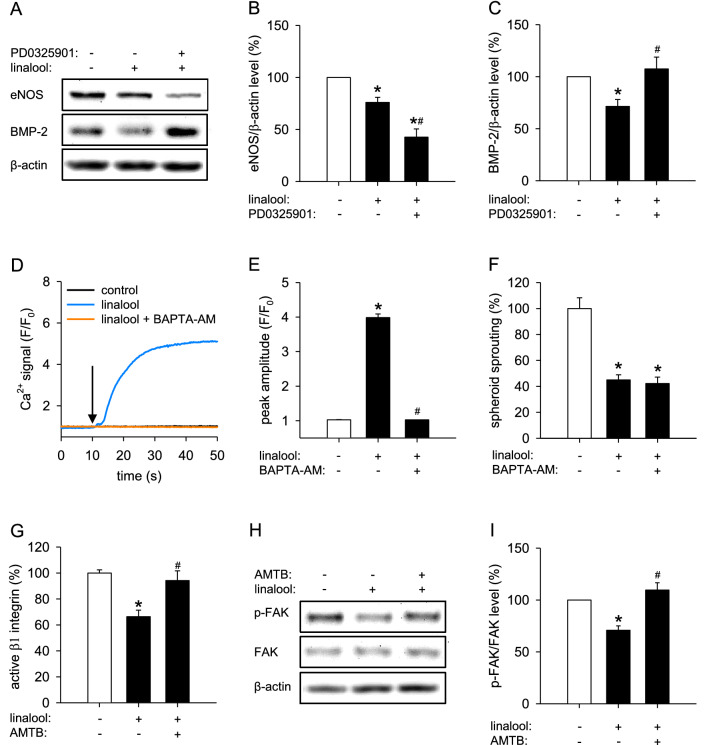
Linalool downregulates BMP-2 by phosphorylating ERK and inhibits β1 integrin/FAK signaling by activating TRPM8. **a** Western blot of eNOS, BMP-2 and β-actin expression in HDMECs, which were pretreated for 2 h with or without 1 µM PD0325901 and then exposed for 30 min to 0 or 2 mM linalool. **b**, **c** Expression levels of eNOS/β-actin (**b**) and BMP-2/β-actin (**c**) (in % of 0 mM linalool) as assessed by western blot (*n* = 4 independent experiments). **d** Ca^2+^ signal (*F*/*F*_0_) in HDMECs, which were pretreated with or without 2.5 µM BAPTA-AM (a selective Ca^2+^ chelator) for 2 h, loaded with Fluo-4 AM and then stimulated with 0 or 2 mM linalool. The onset of stimulation is indicated by a black arrow. **e** Peak amplitude (*F*/*F*_0_) of Ca^2+^ signal in HDMECs, which were pretreated with or without BAPTA-AM, loaded with Fluo-4 AM and then stimulated with vehicle or linalool. **f** Sprouting (in % of 0 mM linalool) of HDMEC spheroids, which were treated for 24 h with 0 or 2 mM linalool in the presence or absence of 2.5 µM BAPTA-AM, as assessed by the spheroid sprouting assay (*n* = 10). **g** Active β1 integrin (in % of 0 mM linalool) in HDMECs, which were pretreated for 2 h with or without 5 µM AMTB and then exposed for 30 min to 0 or 2 mM linalool, as assessed by flow cytometry (*n* = 3). **h** Western blot of p-FAK, FAK and β-actin expression in HDMECs, which were pretreated for 2 h with or without 5 µM AMTB and then exposed for 30 min to 0 or 2 mM linalool. **i** Expression levels of pFAK/FAK (in % of 0 mM linalool) as assessed by western blot (*n* = 4 independent experiments). Means ± SEM. *P < 0.05 vs. 0 mM linalool. ^#^*P* < 0.05 vs. 2 mM linalool

It has been reported that phosphorylated ERK translocates into the nucleus and regulates hundreds of substrates, many of which participate in key physiological processes, such as cell proliferation, migration, survival and death [30]. To identify the downstream effectors of ERK that contribute to the anti-angiogenic effects of linalool, we analyzed the expression of 11 proteins, which are crucially involved in angiogenesis, in vehicle- and linalool-treated HDMECs by western blot. These proteins included BMP-2, CDK4, CDK6, CDK9, COX-2, eNOS, ICAM-1, p21, RhoA, SIRT1 and VEGFA. Among them, the expression levels of eNOS and BMP-2 were significantly downregulated by linalool, while COX-2 was upregulated in linalool-treated HDMECs (Fig. S4a, b). To further determine whether linalool inhibits eNOS and BMP-2 expression through activating ERK, we examined the expression levels of the two proteins in HDMECs that were treated with vehicle or linalool in the presence or absence of PD0325901. Western blot analyses showed that the linalool-induced downregulation of BMP-2, but not eNOS, is completely reversed by ERK inhibition (Fig. 8a–c). These findings indicate that linalool-induced ERK phosphorylation causes BMP-2 downregulation, which mediates the anti-angiogenic effect of linalool.

### Downstream anti‐angiogenic mediators of linalool‐induced TRPM8 activation

Our above findings showed that linalool induces TRPM8 activation and consequently triggers Ca^2+^ influx. To investigate whether the anti-angiogenic effect of linalool is dependent on TRPM8-mediated calcium signaling, the selective Ca^2+^ chelator BAPTA-AM was used in the spheroid sprouting assay. For this purpose, linalool-exposed HDMEC spheroids were treated with a concentration of 2.5 µM BAPTA-AM, which was non-cytotoxic (Fig. S1d), but completely inhibited linalool-induced Ca^2+^ influx (Fig. 8d, e). By this, we could demonstrate that BAPTA-AM does not affect linalool-suppressed spheroid sprouting (Fig. 8f). This indicates that linalool inhibits angiogenesis independently of the pore function of TRPM8.

Genova et al. reported that TRPM8 inhibits EC adhesion through inactivation of β1 integrin/FAK signaling [8]. We therefore analyzed β1 integrin activity and FAK phosphorylation in HDMECs that were treated with vehicle or linalool in the presence or absence of AMTB by flow cytometry and western blot, respectively. We found that treatment with linalool causes a 34% decrease in β1 integrin activity and a 30% reduction in FAK phosphorylation, which could be totally reversed by TRPM8 blockade (Fig. 8g–i). These findings indicate that linalool-induced TRPM8 activation inhibits β1 integrin/FAK signaling, which may explain the anti-angiogenic effect of linalool.

## Discussion

Linalool is a naturally occurring monoterpene alcohol commonly found as a major component of essential oils from many aromatic plants. There are two enantiomers of linalool, i.e., (3*S*)-(+)-linalool and (3*R*)-(−)-linalool, which differ in their distribution in plant species, fragrance and biological properties [31]. The subject of this study was (3*R*)-(−)-linalool, because this enantiomer is more common in nature [15]. Given the fact that in many studies the enantiomeric identity of linalool is not specified, we herein use the term ‘linalool’ for both enantiomers.

Linalool possesses a broad spectrum of biological activities. An increasing number of studies reported anticancer effects of this phytochemical in vitro and in vivo. This action of linalool has been mainly attributed to its anti-proliferative and cytotoxic effects on different types of tumor cells through the generation of oxidative stress, induction of cell cycle arrest and suppression of the epithelial-mesenchymal transition (EMT) process [32–36]. In contrast, the effects of linalool on angiogenesis, which is essential for tumor growth and metastasis, remain elusive. In the present study, we now demonstrate a potent inhibitory effect of linalool on the angiogenic activity of ECs, which may markedly contribute to the anticancer activity of the compound.

Angiogenesis is a complex multi-step process, which includes EC proliferation, migration and tube formation [37]. To investigate which steps of this process are affected by linalool treatment, we performed a panel of in vitro angiogenesis assays. We found that linalool exerts pleiotropic anti-angiogenic effects on HDMECs, including the inhibition of HDMEC proliferation, migration, tube formation and spheroid sprouting. For this purpose, we used rather high linalool doses of 1 and 2 mM, which, however, did not affect the viability of the cells. In this context, it should be considered that linalool exhibits a poor solubility in culture medium and a low in vivo bioavailability. Accordingly, we used DMSO to increase the solubility of the compound in our in vitro assays.

In a next step, we investigated the effects of linalool on angiogenesis in an ex vivo rat aortic ring assay, which is based on the capacity of aortic rings to form new vessels in Matrigel, allowing the analysis of EC proliferation, migration, tube formation, microvessel branching and maturation [38]. Importantly, this assay closely mimics an in vivo environment. In fact, the angiogenic sprouting from aortic rings occurs in the presence of smooth muscle cells, pericytes, macrophages and fibroblasts [38]. In addition, the native ECs of aortic rings have not been modified by repeated passages and are quiescent at the time of explantation [39]. Accordingly, the aortic ring assay is widely used to evaluate the efficiency of pro- and anti-angiogenic compounds. In line with our HDMEC culture experiments, linalool significantly suppressed the vascular sprouting from rat aortic rings. Moreover, we found that linalool significantly reduces the microvessel density of Matrigel plugs 7 days after implantation into BALB/c mice when compared to controls. Notably, in this assay we analyzed the density of CD31-positive microvessels by means of immunohistochemistry. This parameter is more accurate compared to the measurement of plasma volume and hemoglobin mass in plugs, which crucially depends on the plug size [40]. Taken together, our findings demonstrate that linalool exhibits a potent anti-angiogenic activity, which is reproducible in several experimental in vitro and in vivo settings using ECs of different species.

To gain further insights into the mechanisms underlying the anti-angiogenic action of linalool, we examined the effects of the compound on two key angiogenesis-related signaling pathways, i.e., PI3K/AKT/mTOR and Raf/MEK/ERK. In contrast to previous studies reporting that linalool inhibits AKT/mTOR signaling in human oral cancer OECM 1 cells and hepatoma HepG2 cells [23, 41], we did not detect a suppression of AKT phosphorylation in linalool-treated HDMECs. This indicates that the compound acts via different mechanisms in malignant tumor cells and benign ECs. Of interest, we observed that linalool markedly increases the phosphorylation of ERK and inhibition of MEK/ERK signaling by PD0325901 largely reverses linalool-suppressed spheroid sprouting. This is an unexpected finding considering the fact that ERK signaling is widely accepted to promote EC survival and angiogenesis [42], although there are several studies indicating that ERK activation induces cell cycle arrest and apoptosis [5, 7].

Furthermore, we showed that linalool-induced intracellular ATP reduction and TRPM8 activation, but not ROS formation, contribute to the anti-angiogenic effect of linalool. Of interest, the intracellular ATP reduction promoted ERK phosphorylation. This view is supported by our results that (i) ATP supplementation completely reverses linalool-increased phosphorylated ERK and (ii) the inhibition of ERK phosphorylation has no effects on the reduction of intracellular ATP. In contrast, previous studies reported that extracellular ATP stimulates ERK signaling in several types of cells [43–45] and ATP depletion induced by anoxia reduces ERK phosphorylation [46]. These contradictory findings suggest a complex regulatory mechanism of ERK activation dependent on the intracellular level of ATP. As observed in our study, a slight intracellular ATP reduction may serve as a stimulus for the activation of ERK. On the other hand, the phosphorylation of ERK may not be possible under complete ATP deprivation due to the lack of the phosphate donor. The precise mechanism of how a slight intracellular ATP reduction causes ERK phosphorylation needs to be further elucidated. However, it is already known that the inhibitors of mitochondrial complex I rotenone and metformin, which reduce intracellular ATP levels, enhance ERK phosphorylation by promoting the dimerization of the kinase suppressor of Ras (KSR) with Raf, which catalyzes MEK phosphorylation [47]. Since linalool is also a mitochondrial complex I inhibitor [28], we therefore assume that it may induce ERK phosphorylation through regulating KSR-Raf dimerization.

In addition, we found that TRPM8 blockade has no influence on linalool-triggered ATP reduction and ERK phosphorylation. Moreover, ERK inhibition did not affect TRPM8 activity in linalool-treated HDMECs. These results demonstrate that linalool-induced TRPM8 activation is independent on intracellular ATP reduction and ERK phosphorylation. This view is further supported by our observation that linalool activates TRPM8 within seconds, while the linalool-induced phosphorylation of ERK requires a longer time span.

Finally, we investigated the mechanism of how linalool-induced ERK phosphorylation inhibits angiogenesis. By performing a western blot-based small-scale expression screening of angiogenesis-regulatory proteins, we found that BMP-2 is downregulated by linalool-induced ERK phosphorylation. BMP-2 is a member of the transforming growth factor (TGF) superfamily. It has been reported to promote proliferation, migration and tube formation of several types of ECs [48–50]. In vivo, BMP-2 enhances angiogenesis in the mouse sponge assay and stimulates neovascularization in tumors formed from A549 and MCF-7 cells [48, 49]. Notably, several studies demonstrated that BMP-2 acts upstream of ERK [48, 51, 52]. On the other hand, it is well known that phosphorylated ERK translocates into the nucleus, where it regulates different transcription factors [30]. Therefore, it is reasonable that BMP-2 can also be influenced by linalool-induced ERK phosphorylation. In fact, previous studies have already shown that different compounds, including wedelolactone [53], fucoidan [54], imperatorin and bergapten [55], induce ERK phosphorylation in human mesenchymal stem cells or osteoblasts, which, in turn, regulates BMP-2 expression. Accordingly, it may be assumed that the herein observed BMP-2 downregulation is induced by linalool-promoted ERK phosphorylation, and that this mediates the anti-angiogenic effect of linalool. We also demonstrated that the activation of TRPM8 channels triggered by linalool blocks β1 integrin/FAK signaling. These results confirm the observation of Genova et al. that TRPM8 inhibits EC migration via a non-channel function by trapping Rap1 intracellularly and consequently impairing integrin activation [8]. Hence, this ion channel is a promising novel target for anti-angiogenic therapy.

**Fig. 9:**
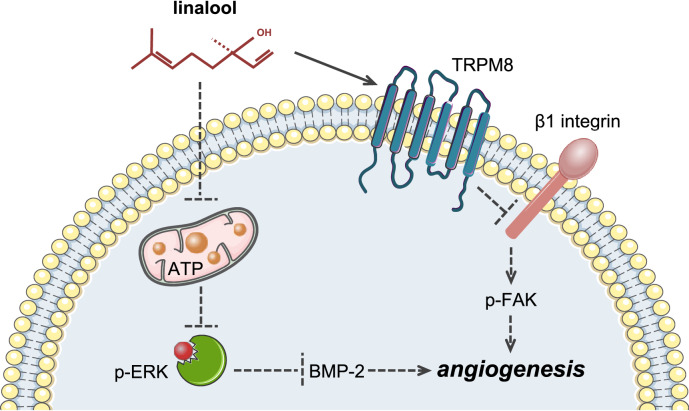
Schematic diagram of molecular mechanisms underlying the inhibitory effect of linalool on angiogenesis

In summary, the present study demonstrates for the first time that linalool acts as an angiogenesis inhibitor by targeting multiple independent angiogenesis-related mediators (Fig. 9). This could be a major advantage of linalool compared to highly selective anti-angiogenic agents, because this pleiotropic action profile may prevent cellular escape mechanisms. Accordingly, linalool may represent a promising therapeutic agent or a lead compound for the future treatment of angiogenesis-related diseases. However, it should be considered that, like other phytochemicals, linalool exhibits a low in vivo bioavailability. Hence, it will be necessary to develop sophisticated carrier systems and novel derivatives or prodrugs to improve the pharmacokinetic properties of the compound. Rapid progress in modern drug design and nanotechnology may markedly contribute to achieve this in the future.

## Supplementary Information

Below is the link to the electronic supplementary material.

Supplementary Material 1 (DOCX 777 kb)
