# Supplementary material for: Linalool inhibits the angiogenic activity of endothelial cells by downregulating intracellular ATP levels and activating TRPM8
Source: Angiogenesis. 2021 Mar 2;24(3):613–30. doi: 10.1007/s10456-021-09772-y (PMC8292279; doi:10.1007/s10456-021-09772-y)
Supplement: Supplementary file 1 — Supplementary Material 1 (DOCX 777 kb) [file 10456_2021_9772_MOESM1_ESM.docx]

**Supplementary Data**

**Linalool inhibits the angiogenic activity of endothelial cells by downregulating intracellular ATP levels and activating TRPM8**

Vivien Becker^1^, Xin Hui^2^, Lisa Nalbach^1^, Emmanuel Ampofo^1^, Peter Lipp^2^, Michael D. Menger^1^, Matthias W. Laschke^1^, Yuan Gu^1^*

^1^Institute for Clinical & Experimental Surgery, Saarland University, 66421 Homburg/Saar, Germany

^2^*Molecular Cell Biology, Research Center for Molecular Imaging and Screening, Medical Faculty, Saarland University, 66421 Homburg/Saar, Germany*

**^*^Corresponding author:**

Yuan Gu, Ph.D.

Institute for Clinical & Experimental Surgery

Saarland University

66421 Homburg/Saar

Germany

phone: +49 6841 162 6368

fax: +49 6841 162 6553

e-mail: yuan.gu@uks.eu


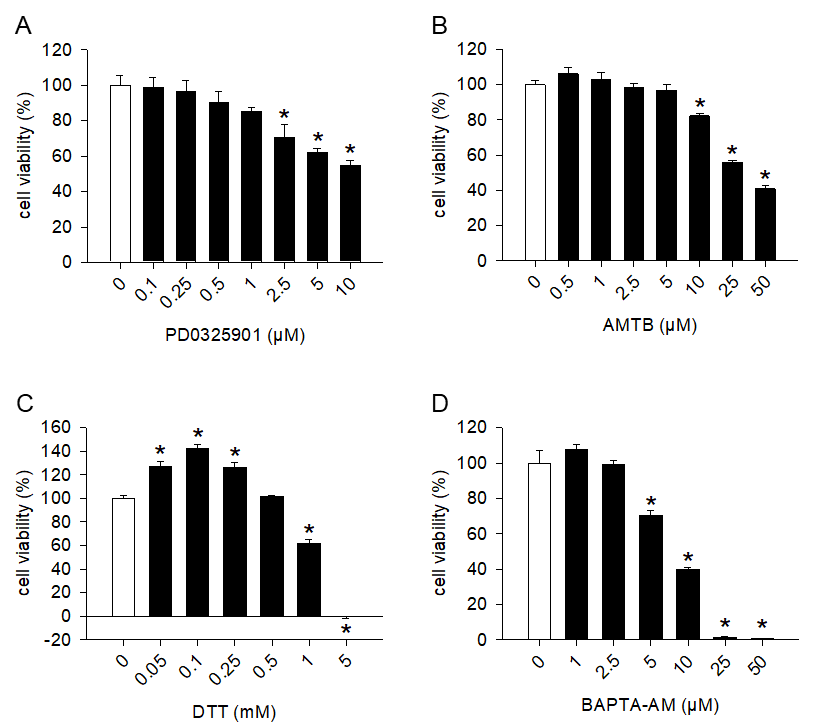


Fig. S1. Effects of PD0325901, AMTB, DTT and BAPTA-AM on HDMEC viability. A-D: Viability (in % of 0 µM or 0 mM) of HDMECs, which were exposed for 24 h to serial dilutions of PD0325901 (A), AMTB (B), DTT (C) or BAPTA-AM (D) as assessed by the WST-1 assay (n = 4). Means ± SEM. *P < 0.05 vs. 0 µM or 0 mM.


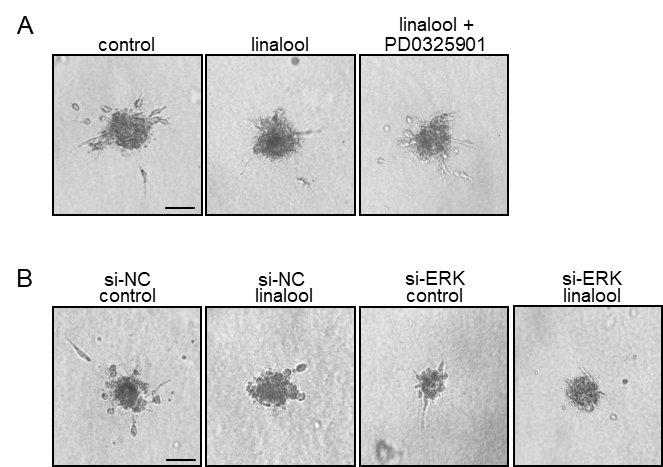


Fig. S2. Representative images of the spheroid sprouting assays in Fig. 5. A: Phase-contrast microscopic images of HDMEC spheroids, which were treated for 24 h with 0 (control) or 2 mM linalool in the presence or absence of 1 µM PD0325901. B: Phase-contrast microscopic images of HDMEC spheroids, which were transfected for 48 h with si-NC or si-ERK and then treated for 24 h with 0 (control) or 2 mM linalool. Scale bars 70 µm.


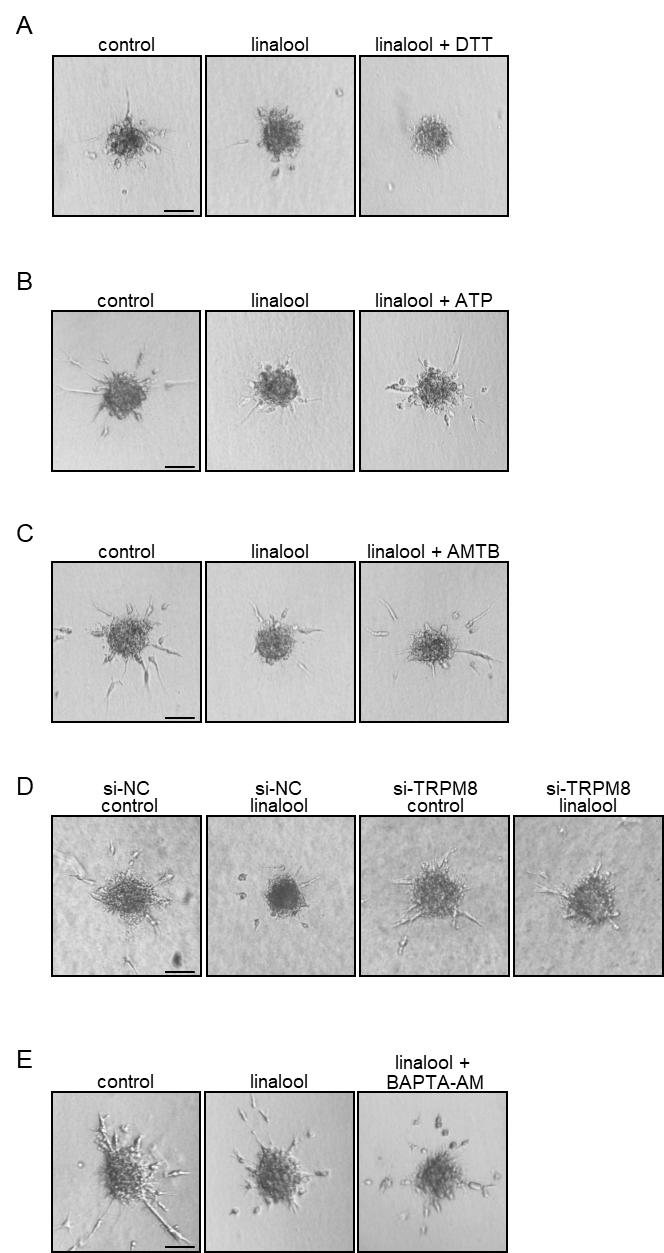


Fig. S3. Representative images of the spheroid sprouting assays in Figs. 6 and 8. A-C: Phase-contrast microscopic images of HDMEC spheroids, which were treated for 24 h with 0 (control) or 2 mM linalool in the presence or absence of 0.5 mM DTT (A), 1 mM ATP (B) or 5 µM AMTB (C). D: Phase-contrast microscopic images of HDMEC spheroids, which were transfected for 48 h with si-NC or si-TRPM8 and then treated for 24 h with 0 (control) or 2 mM linalool. E: Phase-contrast microscopic images of HDMEC spheroids, which were treated for 24 h with 0 (control) or 2 mM linalool in the presence or absence of 2.5 µM BAPTA-AM. Scale bars 70 µm.


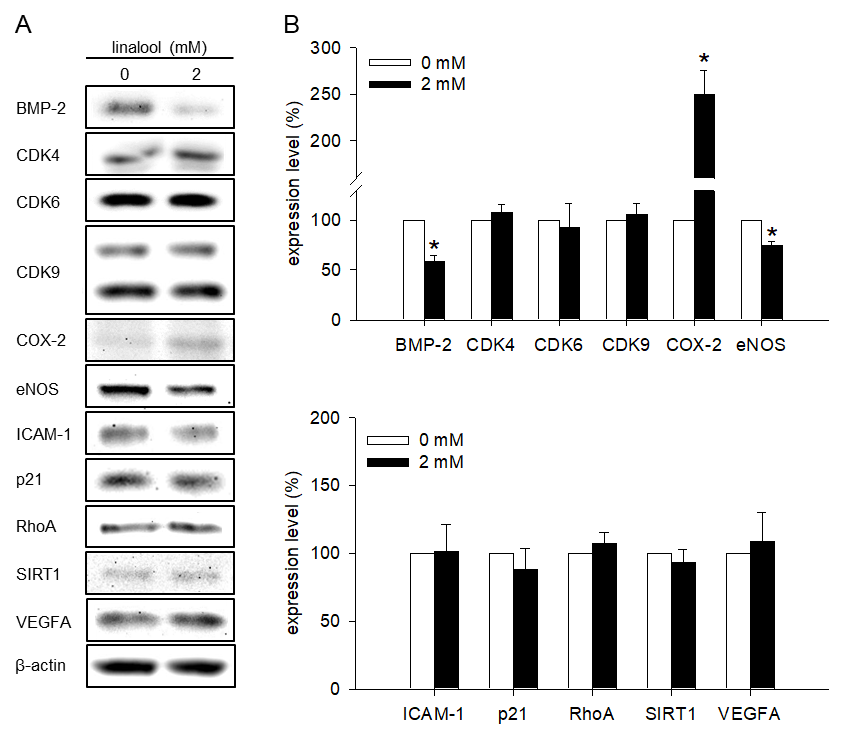


Fig. S4. Effects of linalool on the expression of several angiogenesis-related proteins. A: Western blot of BMP-2, CDK4, CDK6, CDK9, COX-2, eNOS, ICAM-1, p21, RhoA, SIRT1, VEGFA and β-actin expression in HDMECs, which were exposed for 30 min to 0 or 2 mM linalool. B: Expression levels of each protein corrected by β-actin (in % of 0 mM) as assessed by Western blot (n = 3-4 independent experiments). Means ± SEM. *P < 0.05 vs. 0 mM.
